# Supplementary material for: Association between oral microbiome and seven types of cancers in East Asian population: a two-sample Mendelian randomization analysis
Source: Front Mol Biosci. 2023 Nov 21;10:1327893. doi: 10.3389/fmolb.2023.1327893 (PMC10702768; doi:10.3389/fmolb.2023.1327893)
Supplement: Supplementary file 1 [file DataSheet1.ZIP › Supplementary Materials/MR plots for tongue/tongue═╝/Ovarian cancer/pheno.1057_to_ovarian cancer_forest.pdf]

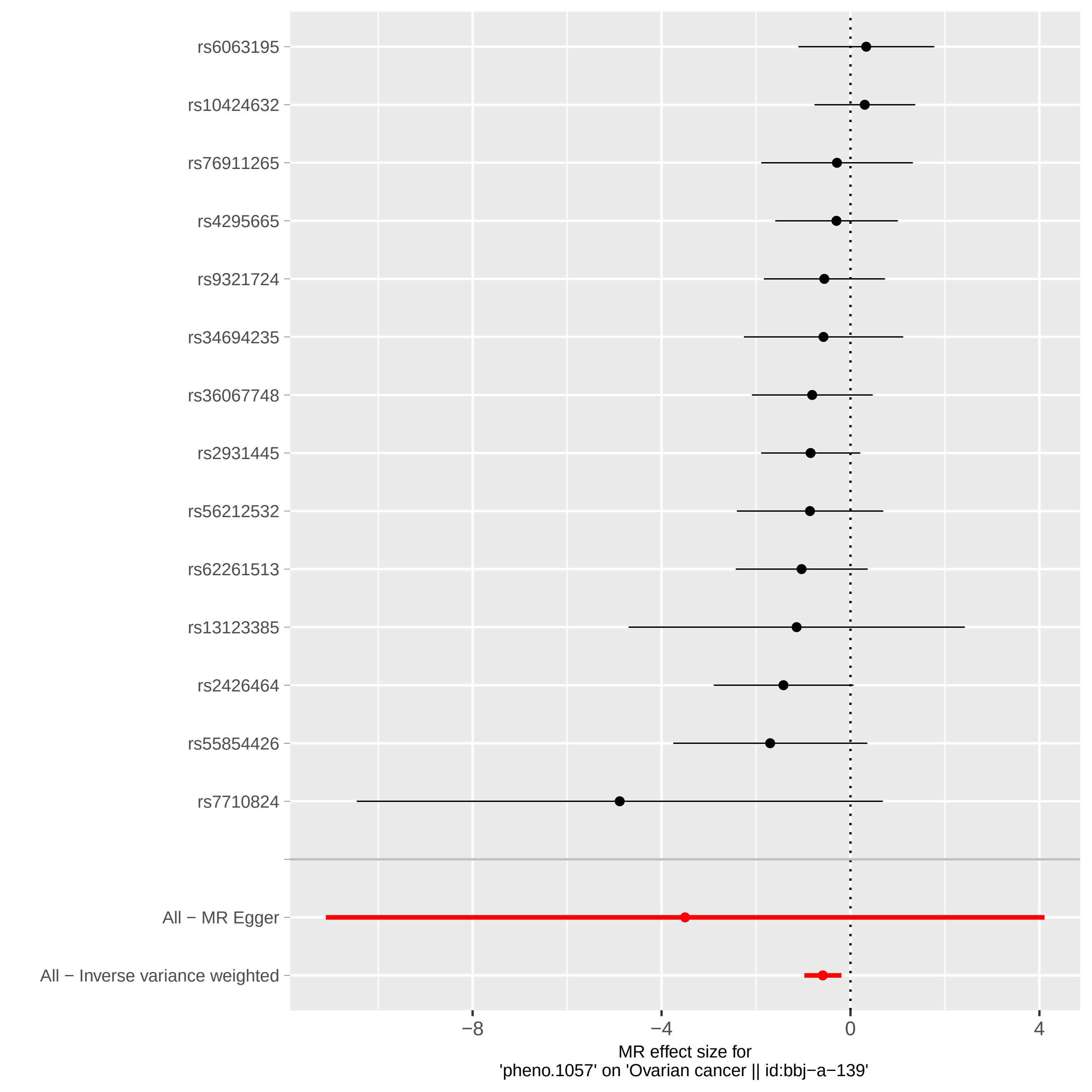
rs6063195
rs10424632
rs76911265
rs4295665
rs9321724
rs34694235
rs36067748
rs2931445
rs56212532
rs62261513
rs13123385
rs2426464
rs55854426
rs7710824
All − MR Egger
All − Inverse variance weighted
−8
−4
0
4
MR effect size for
'pheno.1057' on 'Ovarian cancer || id:bbj−a−139'
